# Prevalence and determinants of health literacy among the adult population of Qatar

**DOI:** 10.3389/fpubh.2023.1278614

**Published:** 2023-11-16

**Authors:** Salma Ahmed, Vahe Kehyayan, Mariam Abdou, Iheb Bougmiza

**Affiliations:** ^1^Department of Community Medicine, Hamad Medical Corporation, Doha, Qatar; ^2^College of Business Management, University of Doha for Science and Technology, Doha, Qatar; ^3^Department of Community Medicine, Primary Healthcare Corporation, Doha, Qatar

**Keywords:** health literacy, health knowledge, prevalence, determinants, Middle East, public health, questionnaire

## Abstract

**Introduction:**

Health literacy (HL) is both a direct determinant and a mediator of health outcomes. Research on the prevalence and determinants of HL in terms of its functional, communicative, and critical domains is scarce in the state of Qatar and its surrounding regions. Thus, this study aims to fill the knowledge gap in this area, estimate the levels of functional, communicative, and critical health literacy among the general adult population, and identify its determinants in the state of Qatar.

**Methods:**

An analytical cross-sectional study with a disproportionate stratified random sampling technique was conducted in 2022. A representative sample of phone numbers was obtained from the Cerner database at Hamad Medical Corporation and approached via well-trained data collectors. A socio-demographic and health-relevant factor questionnaire and the validated All Aspects of Health Literacy scale (AAHLS) were used to collect the data on functional, communicative, and critical HL and their determinants. Descriptive analysis, independent sample *t*-test, ANOVA, and linear regression were used and yielded the outcomes on HL levels as low, adequate, and high in percentages and the HL determinants.

**Results:**

A total of 770 participants were included. The study found that 41.5%, 29.3%, and 29.2% of them have adequate, high, and low overall HL levels consecutively. People who participated in the study are older adult, are of Arabic ethnicity, are of Qatari ethnicity, have a lower level of education, have close relatives with a lower level of education, have a lower income, are non-migrants, are not living within a family, sought medical care within the last week, and who do not know if they have a chronic disease or do not have lower overall HL levels compared to the other groups. After linear regression analysis, only the participant's level of education and “last time sought medical care within last week” variable predict the overall HL score.

**Conclusion:**

Almost half of Qatar's adult population has an adequate HL level, comparable to the HL levels in other regions, despite the limitation in comparison due to variation in context and the HL measurement tools used. The possible determinants are amenable factors to focus on while designing HL interventions and providing healthcare.

## 1 Introduction

Health literacy (HL) is defined as “the achievement of a level of knowledge, personal skills, and confidence to take action to improve personal and community health by changing personal lifestyles and living conditions” (1). Several HL competencies have been identified in the literature resulting from primary studies and other theoretical analyses. Personal skills have been identified as HL competencies and have been categorized as functional, interactive, and critical HL (2). Functional HL describes basic-level skills as sufficient for individuals to obtain relevant health information, such as health risks and how to use the healthcare system, and apply that knowledge to a range of prescribed activities. The concepts of interactive and critical health literacies are linked to contemporary health promotion and health consumer engagement models, where HL is viewed as a personal and population asset providing greater autonomy and control over health decision-making (1). Nutbeam and Lloyd describe interactive HL as more advanced literacy skills that enable individuals to extract health information and derive meaning from the different forms of communication, apply new information to changing circumstances, and engage in interactions with others to extend the information available and make decisions (1). Furthermore, critical HL refers to the most advanced literacy skills that can be applied to critically analyze information from a wide range of sources and information relating to a greater range of health determinants (1).

Measuring and comparing HL levels among different populations is challenging because of variations in the measurement tools used, variations among populations in terms of social and cultural contexts, and variations in health systems (3). In addition, inherent disparities in the social determinants of health have a direct effect on people's HL (3). The results of a systematic review of the prevalence of low HL in the European Union member states showed that the pooled prevalence of low HL ranged from 27% (95% CI: 18–38%) to 48% (95% CI: 41–55%) depending on the literacy assessment method applied (4). In the United States (US), the weighted prevalence of low HL was 26% (95% CI: 22–29%) and marginal HL was 20% (95% CI: 16–23%) (5). In southeast Asia, the overall prevalence of low HL varied considerably, that is, from 1.6% to 99.5%, with a mean of 55.3% (95% CI: 35.1–75.6%) (6). An integrative review of HL research in the Eastern Mediterranean Region (EMR) showed that the levels of HL were similar to those for Europe and the United States (7). A study in Turkey found that 80.7% of the participants had relatively low levels of HL (8). A study in the United Arab Emirates found that over 60% of the population surveyed possessed inadequate HL (9). In a national survey in Saudi Arabia, 46% of the respondents were classified as having low HL (10). In Bahrain, a national survey using the All Aspects of HL Scale (AAHLS) showed that the mean item scores of the survey subscales were higher for empowerment, followed by communicative HL, indicating confidence in these areas compared to critical and functional HL which had lower scores (11).

Several factors have been associated with HL levels. People with higher levels of educational attainment, better jobs, and higher incomes tended to have better access to health information and resources with which to act (12). Poor social and economic conditions were consistently associated with lower HL (12). The strongest association was found between high educational attainment and high HL. Low income, poor occupation, and specific race/ethnicity were also consistently associated with low HL (12). HL was also likely distributed through family and social networks (41). For instance, a person who makes his or her literacy skills available to others, on a formal or informal basis, helps them to accomplish specific literacy purposes; or in a healthcare context, the person helps others to understand medical information by reading or writing information for them for improved interactions with healthcare professionals (13). In European countries with the lowest prevalence of low HL, people have higher levels of education and higher socioeconomic status, which are important factors in HL (4). However, older age is reported to be associated with a higher risk of low HL. Differing cultural and educational backgrounds between patients and healthcare providers may result in different attitudes and beliefs, influencing HL and impairing access to healthcare services (4). The main drivers and strongest associations with low HL in refugees are the lack of knowledge of the healthcare services of the host country, different cultural conceptions, and language barriers (14). In Southeast Asia, the most common factors associated with low HL were education, age, income, and socioeconomic background (6). In the US, the prevalence of low HL was associated with levels of education, ethnicity, and age (5). In general, in EMRO, older adults, individuals with either no or low levels of education, and those residing in rural areas with lower socioeconomic status had lower HL (7). In addition, female individuals, compared to male individuals, had lower HL levels (7). In Iran, monthly income, educational level, and spouse's educational level were significantly associated with HL score levels in older adults (15). In Saudi Arabia, low HL was associated with older age groups, certain country regions, having been formerly married, lower levels of education, lower levels of income, and having sought healthcare exactly three times in the last year (10). In Bahrain, study participants aged < 30 years, who were female individuals, who were married, who were pursuing/completed a Master's program, who were employed, and whose self-rating of health was excellent had higher overall scores (11). In a Palestinian study exploring HL among university students, the authors found that higher scores were significantly associated with having a father with a higher level of education, having a higher frequency of medical checkups, having higher self-reported health status, and consulting a higher number of sources for health-related information (16). Moreover, male students scored significantly higher than female students (16).

Inadequate HL has adverse consequences, including a decreased use of screening and preventive services and decreased compliance with treatments (17). These consequences result in the increased use of emergency services, increased hospitalizations, and, ultimately, higher mortality rates (17). It is also evident that low HL is significantly associated with higher all-cause mortality in patients with heart failure (18).

The concept of HL is well established in Qatar and has been the focus of policymakers and healthcare providers (19, 20). However, while primary research in the field of HL has taken place in numerous countries such as Canada, the United States, and several European countries, in Qatar, it has been limited to specific areas of HL such as mental health, pharmacy, and diabetes and certain populations such as adolescents and pregnant women (19–24). While HL is both a direct determinant and a mediator of health outcomes, research on its functional, communicative, and critical domains is lacking in Qatar. Moreover, an integrative review of HL research in the Eastern Mediterranean Region (EMR) found that most HL research in the region addressed HL among patients in clinical settings, and little was known in the general populations (7). Furthermore, most studies focused on functional HL without mentioning the interactive/communicative and critical HL types despite their importance as empowering skills (7). The specific types of HL, such as mental health, oral, and e-HL, were the focus of research in the EMR rather than general HL (7). Thus, with this study, we intend to fill the knowledge gap on the general adult population's HL in Qatar and its surrounding regions. Therefore, the purpose of this study is to measure the level of HL and its determinants in the state of Qatar among the general adult population to inform policymakers and healthcare providers about the HL gaps and vulnerable groups to develop priority interventions to improve their HL and consequently to help achieve better health outcomes.

## 2 Materials and methods

### 2.1 Study design and study population

In an analytical cross-sectional study conducted between October 2022 and May 2023, the participants' inclusion criteria were age ≥ 18 years, being from all nationalities, and Arabic or English speakers. The only exclusion criterion was people who were living in Qatar for < 3 months (after June 2022) or planning to permanently leave Qatar in the upcoming 3 months (by January 2023), as their HL level will not have an effect on Qatar's health system.

### 2.2 Study setting and procedures

For better representativeness, disproportionate stratified random sampling by gender (50% men/50% women) was used, as in terms of population distribution, the number of female individuals is less than that of male individuals in Qatar (42). A stratified random digit dialing (RDD) sample was used from a nationally representative sampling frame to choose eligible individuals for the study. A list of mobile phone numbers was obtained from the Cerner database (the official electronic medical records database in Qatar). Participants were selected post-stratification by gender by simple random sampling to have 50% men and 50% women. We assumed that 50% of the population would have low HL.

The study sample size was calculated for 95% confidence levels according to the following equation:


$$\begin{array}{l} \text{n}=\left[\text{Np}\left(1-\text{p}\right)\right]/\left[\text{d}2/\text{Z}21-\alpha /2\times \left(\text{N}-1\right)+\text{p}\times \left(1-\text{ p}\right)\right], \end{array}$$

where:

**N: Target population**, a total of 2,092,854 people of 18 years of age and above as per the last report from the Planning and Statistics Authority by the second quarter of 2021 (25).
**n: Sample population** (The required sample size).
**P: The hypothesized prevalence of low HL** will be put at 50% to yield a maximum sample size.
**D: Acceptable error rate** or absolute precision on either side of the proportion: 5% (0.05).
**Z:** Statistic for an error of 0.05 corresponding to a 95% confidence level = (1.96).
**DEFF:** design effect = (1.00).

The minimum sample size that was estimated based on calculation determined (385) using Open Epi (26). As we used a disproportionate stratified random sampling by gender (men/women), we multiplied the sample by 2 to yield a minimum sample size of 770 (385 men and 385 women). Adjustment for non-response rate: We assumed that the response rate would be 40% after adjustment, and the final sample size to approach for participation was calculated based on the following equation: Final sample size = Effective sample size/(1- non-response rate anticipated) = 770/(1 – 0.6) **=** 1,925 (27).

Due to the COVID-19 pandemic to prevent the risk of infection, and for the feasibility of data collection, which was during the FIFA World Cup 2022 period in Qatar, and since most people were working from home for fewer hours and were expected to have enough time and find it convenient to pick the calls, the data were collected by telephone interviews. The data were collected by 15 volunteer data collectors, who could communicate in both Arabic and English, and all of them have a medical background (6 medical interns, 5 medical students, 1 pharmacist, 1 physiotherapist, 1 dentist, and 1 biomedical engineer).

Ethical approval was obtained from the Institutional Review Board (IRB) at Hamad Medical Corporation, and scientific approval was obtained from the Arab Board of Health Specializations. Verbal informed consent was obtained from the participants and recorded; participation was voluntary. Participants had the right to withdraw from the phone call at any time without any penalties. They were offered the option to not answer if they wished. Confidentiality of the data was assured, and it is kept in a password-safe computer.

### 2.3 Study instruments

A **sociodemographic survey** was developed by the researcher after a thorough literature review containing *personal factors* (age, gender, ethnic group, education, occupation, income, and mother language), *sociodemographic factors* (marital status, spouse's educational level, father's educational level, mother's educational level, region of residence at home country, migration status, living in a nuclear family, and residence area in Qatar). *Other relevant factors include* self-rating of health status, last time seeking medical care, having regular medical checkups, and having chronic conditions. HL was measured by the **All Aspects of HL Scale (AAHLS)**, which was developed by King's College, London (26). It is easy to use at the community level. The scale was constructed to reflect three aspects of HL: functional HL (3 items), communicative HL (3 items), and critical HL (7 items), which includes empowerment (3 items) and other critical HL (4 items). Empowerment questions (EMP) in critical HL items address the empowerment of participants (community and social engagement). The AAHLS is a 3-point Likert scale ranging from “rarely” (0), “sometimes” (1), and “often” (2) for the communicative (COM) HL and critical (CR) HL items. However, the prompt in functional (FUN) HL items were “rarely” (2), “sometimes” (1), and “often” (0), non-applicable (3) added to the 2nd question. It was also used and revalidated in Bahrain, a nearby country quite similar to Qatar in its population, and validated for use even in older population groups. It has adequate reliability (Cronbach's alpha = 0.74) and construct validity; scores of functional subscales were significantly associated with communicative subscale (*r* = 0.393, *P* < 0.001) and critical subscale items (*r* = 0.59, *P* = 0.036) and a significant association was found between communicative and critical subscales (*r* = 0.186, *P* = 0.017) (11). The original English version is open-access and available online (28). The translated validated Arabic version (via semantic translation) in an Iraqi study had been accessed after getting approval from the author (29).

### 2.4 Outcome variables

Functional HL: This corresponds to basic reading and writing skills and basic knowledge of health conditions and health systems. It is a categorical variable measured by three questions with three levels of Likert scale response as per the AAHLS. Communicative HL: This corresponds to communicative and social skills to extract information, derive meaning from different forms of communication, and apply to changing circumstances. It is a categorical variable measured by four questions with three levels of Likert scale response as per the AAHLS. Critical HL: This refers to the advanced cognitive and social skills to critically analyze information and exert greater control over life events and situations relating to individual and community-level wellbeing goals. It is a categorical variable measured by four questions with three levels of Likert scale response as per the AAHLS. Empowerment: This addresses the empowerment of participants (community and social engagement, i.e., information and encouragement to lead healthy lifestyles or quality of life). It is a categorical variable measured by three questions with different response options (28).

### 2.5 Data analysis

The data spreadsheet was coded and entered into a database constructed through SPSS (Statistical Package for the Social Sciences) version 29. Even though the original AAHLS tool has no scoring, **the item scores were computed for all 13 items** in the tool. The lowest possible score is 0 and the highest possible score is 2 for the 3 response items, and the lowest possible score is 0 and the highest possible score is 1 for the 2 response items. For the subscale domains, FUN HL, COM HL, CR HL, and empowerment domains, the lower score is 0, while the maximum score is 7, 6, 12, and 4, respectively. For the total AAHLS, the minimum score is 0, while the maximum score is 24. The level of HL was determined based on the 25th and 75th percentiles: low HL if the mean score was ≤ 25th percentile, adequate HL if the score is between the 25th and 75th percentiles, and high HL if the score was ≥75th percentile. A descriptive analysis for all dependent and independent variables was conducted. For quantitative variables, after testing for normality, using skewness and kurtosis, they were described using the mean and standard deviation (if normally distributed) or median and IQR (if not normally distributed). The qualitative variables were described using frequencies and valid percentages. Bivariate correlation analysis was conducted to test for any correlation between the AAHLS and subscale scores and for the subscale scores with each other to test for construct validity. Moreover, Cronbach's alpha was calculated to test for the AAHLS reliability within our study sample. Student t-test and ANOVA with *post-hoc* Bonferroni test were used to analyze any possible associations between the total scale score and subscale scores of AAHLS with the hypothesized determinants of HL. The 95% CIs was calculated to compare the strength of the association between dependent and independent variables. The significance level for the two-tailed and one-tailed *P-value*s was set at a *P* < 0.05. Simple and multiple linear regression analysis was performed to isolate the relationships between the independent variables and the outcome variables from the effects of one or more confounders.

## 3 Results

### 3.1 Response rate and information on non-respondents

Data collection was done from 16 October 2022 to 12 December 2022. A total of 2,000 participants were approached. Of them, 780 responded, which accounted for a 39% response rate, while 61% did not respond due to the following reasons: refused, no answer, call me later, the number is out of reach, and being non-Arabic non-English speakers. Out of the 780 participants, 10 participants did not complete more than 50% of the survey questionnaire, and the data on them were discarded. The final analysis included 770 participants.

### 3.2 Description of the study population

#### 3.2.1 Personal and sociodemographic factors

As shown in Table 1, the mean age was 43 ± 13 years; the minimum age was 19 years, while the maximum was 100 years. The age group of 31–45 years predominated the sample. The male participants outweighed the female participants by approximately 7%, with a prevalence of 48.7% and 51.3%, respectively. In total, 462 (60.1%) participants stemmed from the Arab ethnic group, 590 (76.7%) held a non-Qatari nationality, and 545 (62.6%) participants have Arabic as the mother language. Socially, 78.7% of the study participants are married. In addition, most of them live with family (82.6%). Geographically, 374 (48.8%) of the study participants reside in Al-Dawhah district.

**Table 1:** The personal and sociodemographic factors among the study participants in Qatar 2022.

**Personal and sociodemographic variables**	**Frequency (valid %)**	***N***
**Age categories**		770
18–30	132 (17.1)	
31–45	369 (47.9)	
46–60	182 (23.6)	
≥ 61	87 (11.3)	
**Gender**		770
Men	375 (48.7)	
Women	395 (51.3)	
**Ethnic group**		769
Arab	462 (60.1)	
Non-Arab	307 (39.9)	
**Nationality**		769
Qatari	179 (23.3)	
Non-Qatari	590 (76.7)	
**Education**		763
No formal education	12 (01.6)	
Primary school	27 (03.5)	
Preparatory school	40 (05.2)	
Secondary school	140 (18.2)	
University graduate	465 (06.5)	
Postgraduate study	79 (10.3)	
**Occupation**		758
Healthcare provider	62 (08.1)	
Housewife	96 (12.6)	
Manual worker	98 (12.9)	
Office worker	379 (49.7)	
Retired	62 (08.1)	
Student	17 (02.2)	
Un-employed	44 (05.8)	
**Income in QAR per month**		769
< 10,000	221 (28.7)	
10,000–30,000	202 (26.3)	
>30,000–50,000	58 (07.5)	
>50,000	36 (04.7)	
Non-applicable	134 (17.4)	
I do not want to answer	118 (15.3)	
**Mother language**		712
Arabic	445 (62.6)	
English	27 (03.8)	
Urdu	38 (05.3)	
Other	202 (28.3)	
**Marital status**		708
Single	126 (16.4)	
Married	547 (78.7)	
Widow	19 (02.5)	
Divorced	16 (02.1)	
**Spouse educational level**		741
Illiterate/No formal education	27 (**0**3.6)	
Primary school	25 (**0**3.3)	
Preparatory school	27 (**0**3.6)	
Secondary school	109 (14.5)	
University graduate/college	356 (47.3)	
Postgraduate	48 (**0**6.4)	
Non-applicable	149 (19.8)	
**Father's educational level**		763
Illiterate/No formal education	158 (20.7)	
Primary school	73 (09.6)	
Preparatory school	66 (08.7)	
Secondary school	160 (21.0)	
University graduate/college	235 (30.8)	
Postgraduate	71 (09.3)	
**Mother's educational level**		761
Illiterate/No formal education	219 (28.8)	
Primary school	85 (11.2)	
Preparatory school	60 (07.9)	
Secondary school	189 (24.8)	
University graduate/College	157 (20.6)	
Postgraduate	51 (06.7)	
**Migration status**		767
Yes	552 (71.8)	
No	215 (28.0)	
**Region of residence in home**		766
**country**		
Urban	384 (49.9)	
Rural	132 (17.2)	
Non-applicable	221 (28.7)	
I do not know	29 (03.8)	
**Living in family**		770
Yes	636 (82.6)	
No	134 (17.4)	
**Residence area in Qatar**		751
AL-Shamal	24 (03.1)	
Al-Khor	26 (03.4)	
Al-Shahaniya	19 (02.5)	
Umm-Salal	50 (06.5)	
AL-Daayen	20 (02.6)	
Al-Dawhah	374 (48.8)	
Al-Rayyan	149 (19.4)	
Al-Wakrah	89 (11.6)	

#### 3.2.2 Health-relevant factors

As shown in Table 2, 419 (54.6%) participants rated their health status as good, while 190 (24.7%) rated it as excellent; 254 (33.1%) participants sought medical care within the last month; 403 (52.4%) have regular medical checkups; and 312 (40.6%) reported having a chronic condition.

**Table 2:** The health-relevant factors among the general adult populations in Qatar in 2022.

**Health-relevant factors**	**Frequency (Valid %)**	***N***
**Self-rating of health status**		766
Poor	23 (03.0)	
Fair/OK	128 (16.7)	
Good	419 (54.6)	
Excellent	190 (24.7)	
I do not know	6 (00.8)	
**Last time sought medical care**		766
Last week	173 (22.5)	
Last month	254 (33.1)	
Last 6 months	218 (28.4)	
Last year	64 (08.3)	
More than a year	57 (07.4)	
**Having regular medical checkups**		769
Yes	403 (52.4)	
No	366 (47.6)	
**Having chronic disease**		767
Yes	312 (40.6)	
No	441 (57.3)	
I do not know	14 (01.8)	

### 3.3 Description of the AAHLS score and subscale scores

The skewness for the functional HL score, critical HL score, empowerment score, and overall score is less than zero, but still, it is not less than (−0.4), is almost normal (30), and, in contrast, is negatively skewed for the communicative HL score (skewness −1.5), as shown in Table 3. On the other hand, the distributions of the overall score and all the subscale scores have a negative kurtosis (Platykurtic, kurtosis < 3), as shown in Table 3.

**Table 3:** Descriptive analysis for the AAHLS and subscale scores among the general adult populations in Qatar 2022.

	**Functional HL**	**Communicative HL**	**Critical HL**	**EMP**	**Overall**
Min–Max	0–7	0–6	0–12	0–4	2–24
Mean (±SD)	3.6 (±1.6)	5.1 (±1.3)	6.7 (±2.6)	2.3 (± 1.2)	15.4 (±3.5)
Median (± IQR)	4.0 (±1.0)	6.0 (±2.0)	7.0 (±4.0)	2.0 (±2.0)	16.0 (±5.0)
Q1	3	4	5	1	13
Q2	4	6	7	2	16
Q3	4	6	9	3	18
Skewness	−0.08	-−1.5	−0.24	−0.18	−0.27
Kurtosis	−0.01	2.0	−0.60	−0.18	−0.13

The AAHLS shows low but acceptable internal reliability and Cronbach's alpha coefficient was 0.51, while it shows a good construct validity, as reflected by the positive correlation between the subscale scores and the overall scale score with a *P* < 0.001, the functional HL score (r = 0.48), communicative HL score (*r* = 0.49), critical HL score (*r* = 0.81), and empowerment score (*r* = 0.476), as shown in Table 4. In addition, there is a positive correlation between the following subscale scores: communicative HL score with critical HL and empowerment HL scores (*r* = 0.16 and 0.13, respectively), and the critical HL score is positively correlated with the empowerment score (*r* = 0.59) at a *P* < 0.001.

**Table 4:** Correlation between the All Aspects of HL Scale and the subscale scores among the general adult population in Qatar 2022.

	**Functional HL score**	**Communicative HL score**	**Critical HL score**	**Empowerment score**	**Overall score**
Functional HL score	1	0.013	0.021	−0.014	**0.482** ^**^
Communicative HL Score	0.013	1	**0.164** ^**^	**0.129** ^**^	**0.488** ^**^
Critical HL score	0.021	**0.164** ^**^	1	**0.590** ^**^	**0.812** ^**^
Empowerment score	−0.014	**0.129** ^**^	**0.590** ^**^	1	**0.476** ^**^
Overall score	**0.482** ^**^	**0.488** ^**^	**0.812** ^**^	**0.476** ^**^	1

^*^Correlation is significant at the 0.05 level (2-tailed).

^**^Correlation is significant at 0.01 level (2-tailed *P*-value).

### 3.4 Prevalence and levels of HL

**Figure 1:**
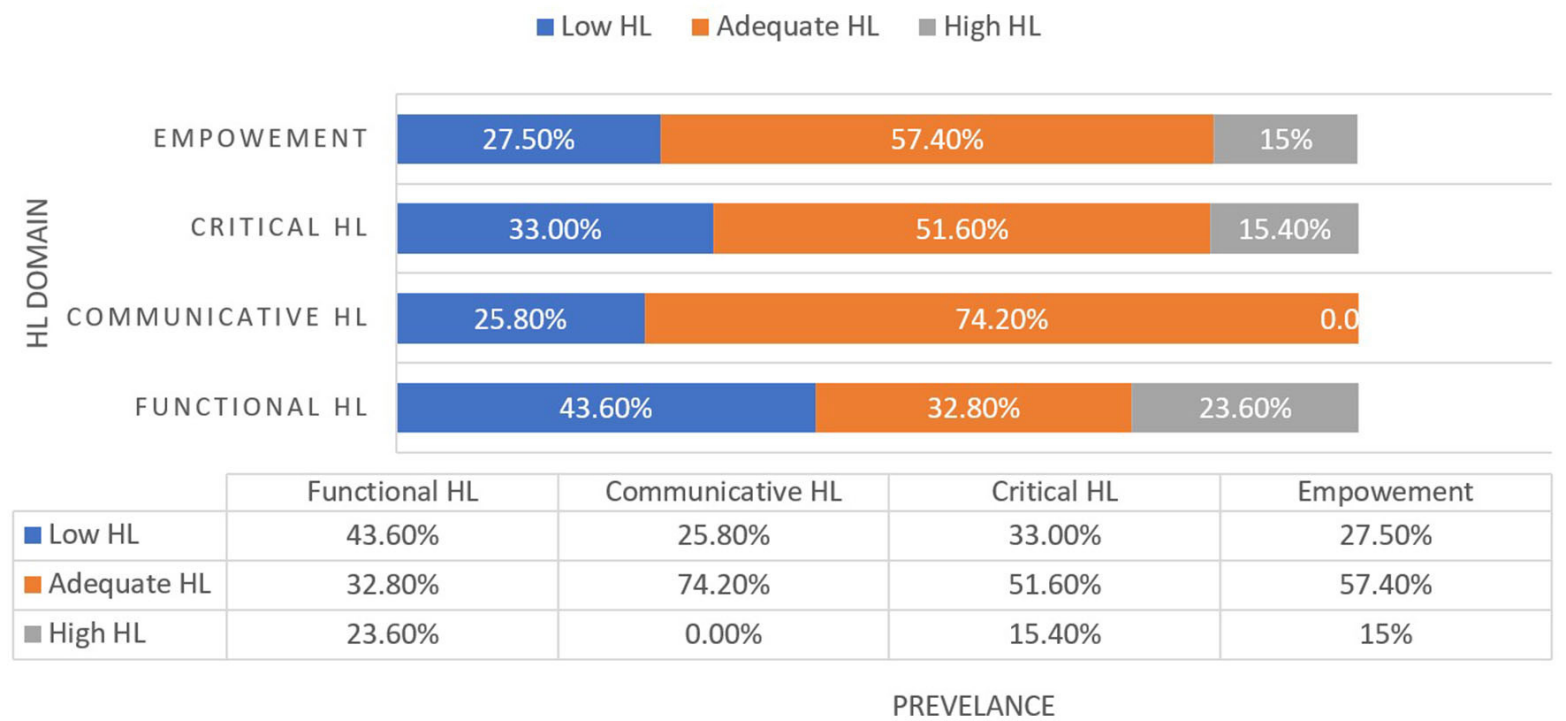
Prevalence of HL competencies among the general adult population in Qatar 2022.

**Figure 2:**
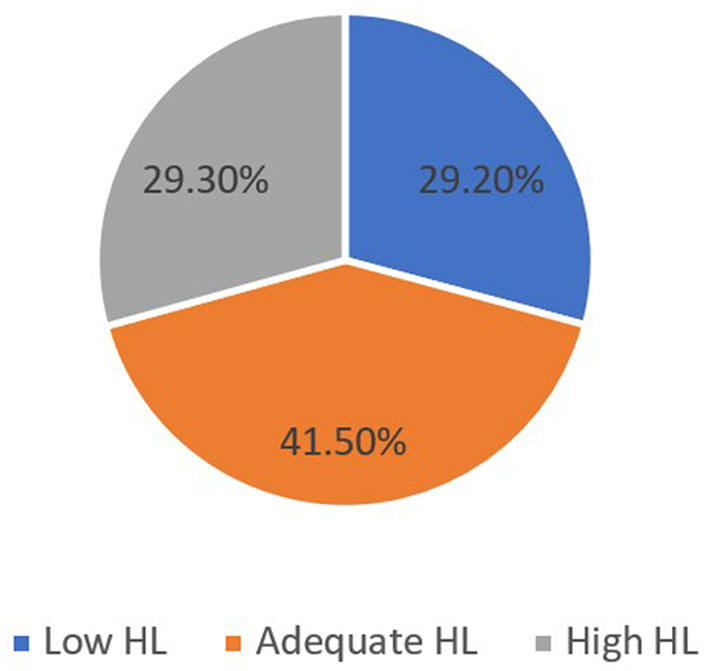
Prevalence of the overall HL among the general adult population in Qatar 2022.

Out of the 770 study participants, 336 (43.6%) have low functional HL, 199 (25.8%) have low communicative HL, 254 (33%) have low critical HL, and 212 (27.5%) have low empowerment, as shown in Figure 1. In addition, 225 (29.2%) have low overall HL, as shown in Figure 2.

### 3.5 Determinants of HL

#### 3.5.1 Determinants of functional HL score

Regarding the personal and sociodemographic variables, in Table 5, the mean functional HL score is strongly associated with age categories, ethnic group, and nationality. Those who are ≥61 years of age have a significantly lower mean functional HL score compared to the middle group of 31–45 years (*P-value*s 0.005). The Arab ethnic group has a significantly lower mean functional HL score than the non-Arab (*P-value* 0.023), and the Qatari nationality has a significantly lower mean functional HL score than the non-Qatari (*P-value* 0.004). In addition, there is a very strong association between the functional HL score and the participant's level of education, spouse's level of education, father's level of education, and mother's level of education; those with no formal education have a significantly lower mean functional HL score compared to those with less educational levels, (*P* ≤ 0.001, 0.002, < 0.001, and < 0.001, respectively). Being non-migrant is very strongly associated with having a lower mean functional HL score than migrants (*P* < 0.001). On the other hand, there is no evidence of an association between the mean functional HL score and the participant's gender, occupation, income, mother language, marital status, living with family, residence area in Qatar, region of residence at home country, and all the health-relevant factors, as shown in Table 6 (all *P-value*s are >0.05).

**Table 5A:** Bivariate analysis of the HL scores and personal and socio-demographic variables among the general adult population in Qatar 2022.

	**FUN HL**		**COM HL**		**CR HL**		**Overall HL**	
	**Mean (**±**SD)**	***P*** **-value**	**Mean (**±**SD)**	***P*** **-value**	**Mean (**±**SD)**	***P*** **-value**	**Mean (**±**SD)**	***P*** **-value**
**Age categories**		**0.005** ^*^		**0.004** ^*^		**0.006** ^*^		**0.010** ^*^
18–30	3.6 (± 1.7)		4.8 (±1.3)		6.7 (±2.6)		15.2 (±3.7)	
31–45	3.8 (± 1.5)		5.0 (±1.4)		6.8 (±2.5)		15.5 (±3.3)	
46–60	3.5 (± 1.7)		5.3 (±1.0)		6.9 (±2.7)		15.7 (±3.5)	
≥61	3.2 (± 1.6)		5.3 (±1.1)		5.8 (±2.7)		14.3 (±3.7)	
**Gender**		0.575		0.176		0.109		0.824
Men	3.7 (± 1.5)		5.1 (±1.2)		6.5 (±2.7)		15.4 (±3.7)	
Women	3.6 (± 1.7)		5.0 (±1.3)		6.8 (±2.5)		15.4 (±3.3)	
**Ethnic group**		**0.023** ^*^		**< 0.001** ^*^	6	0.628		0.415
Arab	3.5 (± 1.6)		5.2 (±1.0)		0.7 (±2.7)		15.5 (±3.5)	
Non-Arab	3.8 (± 1.6)		4.8 (±1.5)		6.6 (±2.5)		15.3 (±3.5)	
**Nationality**		**0.004** ^*^		0.256		**0.049** ^*^		**0.009** ^*^
Qatari	3.3 (± 1.8)		5.2 (± 1.2)		6.3 (±2.6)		14.8 (±3.5)	
Non-Qatari	3.7 (±1.5)		5.0 (±1.3)		6.8 (±2.6)		15.7 (±3.5)	
**Education**		**< 0.001** ^*^		0.313		**< 0.001** ^*^		**< 0.001** ^*^
No formal education	1.8 (± 1.7)		5.0 (±1.1)		5.9 (±2.3)		12.8 (±3.4)	
			5.1 (±1.3)		5.3 (±2.6)		13.6 (±3.5)	
Primary school	3.1 (± 1.5)		5.0 (±1.5)		6.3 (±3.1)		14.1 (±4.1)	
Preparatory school	2.8 (± 2.0)		4.8 (±1.8)		6.0 (±2.6)		14.1 (±3.4)	
Secondary school	3.2 (± 1.6)		5.1 (±1.2)		6.9 (±2.5)		15.8 (±3.2)	
University graduate	3.8 (± 1.5)		5.2 (±1.3)		7.5 (±2.5)		17.0 (±3.8)	
Postgraduate study	4.3 (± 1.6)		5.6 (±0.5)		3.2 (±1.3)		11.6 (±3.6)	
**Occupation**		0.219		0.643		0.630		0.403
Healthcare provider	3.5 (± 1.4)		5.0 (±1.3)		6.5 (±2.7)		15.1 (±3.6)	
Housewife	3.5 (± 1.7)		4.9 (±1.4)		6.4 (±2.5)		14.8 (±3.7)	
Manual worker	3.8 (± 1.6)		5.1 (±1.2)		6.5 (±2.6)		15.3 (±3.3)	
Office worker	3.6 (± 1.6)		5.1 (±1.2)		6.8 (±2.6)		15.5 (±3.5)	
Retired	3.7 (±1.6)		5.2 (±1.3)		6.4 (±2.4)		15.3 (±3.5)	
Student	2.9 (±1.5)		5.1 (±1.2)		7.3 (±2.6)		15.3 (±3.8)	
Un-employed	4.1 (±1.4)		5.1 (±1.0)		7.1 (±2.8)		16.3 (±3.2)	
**Income in QAR per month**		0.232		**< 0.001** ^*^		0.063		**0.002** ^*^
< 10,000 10,000–30,000	3.6 (±1.6) 3.7 (±1.5)		4.8 (±1.5) 5.1 (±1.2)		6.6 (±2.7) 6.9 (±2.8)		14.9 (±3.6) 15.8 (±3.6)	
>30,000–50,000	3.5 (±1.4)		5.3 (±1.1)		7.3 (±2.2)		16.0 (±3.2)	
>50,000	4.1 (±1.5)		5.6 (±0.8)		7.4 (±2.2)		17.1 (±3.4)	
I do not want to answer	3.6 (±1.7)		5.1 (±1.0)		6.3 (±2.5)		15.0 (±3.2)	
**Mother language**		0.401		0.861		0.519		0.852
Arabic	3.7 (±1.6)		5.0 (±1.3)		6.6 (±2.6)		15.3 (±3.6)	
English	3.9 (±1.8)		5.0 (±1.2)		6.9 (±2.2)		15.8 (±3.1)	
Urdu	3.3 (±1.6)		5.2 (±1.3)		7.3 (±3.1)		15.7 (±4.6)	
Other	3.6 (±1.6)		5.1 (±1.2)		6.6 (±2.6)		15.3 (±3.3)	
**Marital status**		0.232		0.643		0.587		0.682
Single	3.8 (±1.5)		4.9 (±1.4)		6.5 (±2.7)		15.3 (±3.7)	
Married	3.6 (±1.6)		5.1 (±1.3)		6.8 (±2.6)		15.4 (±3.5)	
Widow	3.1 (±1.7)		5.2 (±1.0)		6.4 (±2.5)		14.6 (±2.9)	
Divorced	3.6 (±1.8)		5.3 (±1.0)		6.5 (±2.3)		15.5 (±3.7)	

^*^P < 0.05.

**Table 5B:** Bivariate analysis of the HL scores and personal and socio-demographic variables among the general adult population in Qatar 2022.

	**FUN HL**		**COM HL**		**CR HL**		**Overall HL**	
	**Mean (**±**SD)**	***P*** **-value**	**Mean (**±**SD)**	***P*** **-value**	**Mean (**±**SD)**	***P*** **-value**	**Mean (**±**SD)**	***P*** **-value**
**Spouse's educational level**		**0.002** ^*^		0.198		0.170		**0.008** ^*^
No formal education	3.2 (± 1.8)		5.3 (±1.4)		6.1 (±3.2)		14.5 (±4.7)	
Primary school	3.1 (± 1.7)		4.5 (±1.8)		6.7 (±3.0)		14.3 (±4.3)	
Preparatory school	3.0 (± 1.8)		5.4 (±0.8)		6.3 (±3.3)		14.7 (±3.6)	
Secondary school	3.3 (± 1.7)		5.0 (±1.3)		6.5 (±2.3)		14.9 (±3.2)	
University/College	3.7 (±1.6)		5.1 (±1.2)		6.8 (±2.6)		15.6 (±3.4)	
Postgraduate	4.2 (±1.3)		5.1 (±1.3)		7.5 (±2.1)		16.9 (±2.9)	
**Father's educational level**		**< 0.001** ^*^		0.089		0.077		**0.004** ^*^
No formal education	3.1 (±1.7)		5.2 (±1.1)		6.3 (±2.9)		14.7 (±3.7)	
Primary school	3.6 (±1.6)		5.0 (±1.4)		6.2 (±2.4)		14.8 (±3.5)	
Preparatory school	3.5 (±1.7)		4.9 (±1.4)		6.6 (±2.6)		15.0 (±3.3)	
Secondary school	3.7 (±1.5)		4.9 (±1.4)		6.8 (±2.6)		15.4 (±3.5)	
University/College	3.9 (±1.5)		5.1 (±1.3)		7.0 (±2.4)		16.0 (±3.2)	
Postgraduate	3.7 (±1.8)		5.1 (±1.0)		6.8(±2.7)		15.6 (±3.8)	
**Mother's educational level**		**< 0.001** ^*^		0.523		0.054		**0.012** ^*^
No formal education	3.3 (±1.6)		5.2 (±1.1)		6.3 (±2.7)		14.8 (±3.5)	
Primary school	3.7 (±1.7)		5.0 (±1.4)		6.5 (±2.5)		15.3 (±3.4)	
Preparatory school	3.3 (±1.5)		5.0 (±1.3)		6.7 (±2.8)		14.9 (±3.7)	
Secondary school	3.9 (±1.5)		5.0 (±1.3)		7.1 (±2.4)		15.9 (±3.3)	
University/College	3.9 (±1.5)		5.0 (±1.3)		7.0 (±2.4)		15.9 (±3.3)	
Postgraduate	3.6 (±1.9)		5.1 (±1.1)		6.7 (±3.0)		15.2 (±3.9)	
**Migration status**		**< 0.001** ^*^		0.207		0.070		**0.011** ^*^
Yes	3.8 (± 1.5)		5.0 (±1.3)		6.8 (±2.6)		15.6 (±3.5)	
No	3.3 (± 1.6)		5.2 (±1.1)		6.4 (±2.6)		14.8 (±3.4)	
I do not want to answer	5.0 (±1.4)		5.5 (±0.7)				15.5 (±9.2)	
**Region of residence in**		0.199		0.382		0.199		0.260
**home country**								
Urban	3.8 (±1.5)		5.0 (±1.3)		7.0 (±2.5)		15.8 (±3.4)	
Rural	3.7 (±1.6)		5.0 (±1.5)		6.5 (±2.7)		15.1 (±3.9)	
I do not know	3.8 (±1.8)		5.2 (±1.1)		6.8 (±3.2)		15.9 (±3.2)	
**Living in the family**		0.360		0.360		0.061		**0.008** ^*^
Yes	3.7 (±1.6)		5.1 (±1.2)		6.8 (±2.6)		15.5 (±3.4)	
No	3.5 (±1.5)		4.8 (±1.5)		6.3 (±2.8)		14.6 (±3.8)	
**Residence area in Qatar**		0.444		0.581		0.153		0.080
AL-Shamal	3.6 (±1.7)		5.1 (±1.1)		6.0 (±2.9)		14.7 (±3.5)	
Al-Khor	3.7 (±1.2)		5.0 (±1.3)		3.7 (±1.2)		15.7 (±3.5)	
Al-Shahaniya	2.7 (±2.1)		4.6 (±1.9)		2.7 (±2.1)		13.1 (±3.7)	
Umm-Salal	3.4 (±1.5)		5.1 (±1.2)		3.4 (±1.5)		15.4 (±3.7)	
AL-Daayen	3.5 (±1.8)		5.0 (±1.1)		3.5 (±1.8)		14.4 (±3.7)	
Al-Dawhah	3.7 (±1.6)		5.0 (±1.3)		3.7 (±1.6)		15.6 (±3.5)	
Al-Rayyan	3.6 (±1.5)		5.2 (±1.1)		3.6 (±1.5)		15.6 (±3.4)	
Al-Wakrah	3.8 (±1.6)		3.8 (± 1.6)		3.8 (±1.6)		15.3 (±3.4)	

^*^P < 0.05.

**Table 6:** Bivariate analysis of the HL scores and health-relevant factors among the general adult population in Qatar 2022.

	**FUN HL**		**COM HL**		**CR HL**		**Overall HL**	
	**Mean (**±**SD)**	***P*** **-value**	**Mean (**±**SD)**	***P*** **-value**	**Mean (**±**SD)**	***P*** **-value**	**Mean (**±**SD)**	***P*** **-value**
**Self-rating of health status**		0.355		0.543		0.071		0.031^*^
Poor	3.3 (±1.9)		4.8 (±1.4)		7.5 (±2.4)		15.6 (±3.2)	
Fair/OK	3.4 (±1.7)		4.9 (±1.3)		6.4 (±2.8)		14.7 (±3.8)	
Good	3.7 (±1.5)		5.1 (±1.2)		6.7 (±2.5)		15.6 (±3.3)	
Excellent	3.6 (±1.6)		5.1 (±1.3)		6.8 (±2.6)		15.5 (±3.6)	
I do not know	3.2 (±2.7)		4.5 (±1.0)		4.5 (±1.9)		12.2 (±2.5)	
**Last time sought medical care**		0.251				**0.005** ^*^		**0.020** ^*^
Last week	3.5(± 1.6)		5.2 (±1.1)		2.8 (±2.5)		15.6 (±3.4)	
Last month	3.7 (±1.6)		5.0 (±1.2)		6.9 (±2.6)		15.6 (±3.5)	
Last 6 months	3.6 (±1.7)		5.2 (±1.2)		6.8 (±2.6)		15.6 (±3.3)	
Last year	3.4 (±1.7)		4.9 (±1.2)		5.7 (±2.6)		14.1 (±3.7)	
More than a year	4.0 (±1.6)		4.5 (±1.8)		6.2 (±2.8)		14.8 (±4.1)	
**Having regular medical checkups**		0.123		0.123		0.064		0.094
Yes	3.5 (±1.6)		5.2 (±1.2)		6.9 (±2.5)		15.6 (±3.4)	
No	3.7 (±1.6)		4.9 (±1.3)		6.5 (±2.7)		15.2 (±3.6)	
**Having chronic disease**		0.051		0.117		**0.014** ^*^		0.050
Yes	3.5 (±1.7)		5.2 (±1.1)		6.7 (±2.7)		15.3 (±3.7)	
No	3.7 (±1.5)		5.0 (±1.3)		6.8 (±2.5)		15.5 (±3.3)	
I do not know	4.2 (±2.0)		4.5 (±1.6)		4.7 (±1.6)		13.4 (±3.4)	
I do not want to answer	3.0 (±0.0)		4.5(±0.71)					

^*^P < 0.05.

#### 3.5.2 Determinants of communicative HL score

Table 5 shows the relationship between the communicative HL score and the personal and socio-demographic factors. The 18–30 age group has a significantly lower communicative HL score than higher age categories (*P-value* 0.004). The non-Arab ethnic group has a significantly lower communicative HL score compared to Arab, and those whose monthly income is < 10,000 QAR (< 2,740 USD) have a significantly lower communicative HL score compared to higher-income groups and those who do not want to answer the income question (*P* > 0.001). For the remaining individuals, the socio-demographic variable and all four health-relevant variables, shown in Table 6, indicate that there is no evidence of any association with the communicative HL score (*P* > 0.05).

#### 3.5.3 Determinants of ***critical HL score***

As shown in Table 5, people aged 61 years or older have a significantly lower critical HL score compared to younger age groups, and Qatari nationals have a significantly lower critical HL score compared to Non-Qatari (*P-value* 0.006 and 0.049, respectively). Those who are postgraduates and have a university or college level of education have a significantly higher critical HL score than those with low education level (*P* < 0.001). For health-relevant factors, as shown in Table 6, those who reportedly sought medical care within the last week have a significantly lower critical HL score compared to those who sought medical care earlier before the last week (*P-value* 0.005), and those who reported that they do not know if they have a chronic disease or not have a significantly lower critical HL score compared to those who know (*P-value* 0.014). There is no evidence of association with the critical HL score regarding the remaining personal, sociodemographic, and health-relevant factors (*P* > 0.05).

#### 3.5.4 Determinants of total AAHLS score

For total HL, those with a monthly income of < 10,000 QAR (< 2,740 USD) have a significantly lower overall score compared to higher-income groups (*P* < 0.002). In addition, there is strong evidence of an association between the participant's level of education, spouse's level of education, father's level of education, and mother's level of education and the overall score. Those who are university/college graduates and postgraduates have a significantly higher overall score compared to less education level groups (*P* < 0.001, 0.008, 0.004, and 0.012, respectively). While there is no significant difference in the overall score between Arab and non-Arab ethnic groups, Qatari nationals have a significantly lower overall score than non-Qatari (*P-value*s 0.415 and 0.009, respectively). Socially, non-migrants have a significantly lower overall score compared to migrants, and those who do not live with their family have a significantly lower overall score compared to those who live with their family (*P-value* 0.011 and 0.008, respectively). For health-relevant factors, as shown in Table 6, those who last sought medical care 1 year back have a significantly lower overall score compared to those who sought medical care more recently (*P-value* 0.020), and those who do not know how to self-rate their health status have a significantly lower overall score compared to those who know (*P-value* 0.031).

It is worth mentioning that there was no difference in the overall score between male and female individuals, ethnic groups, occupation categories, participant's mother language, marital status, or residence area either in the home country or in Qatar, those who have a regular checkup and those who do not, and those who reported having a chronic disease, do not have it, or even do not know of having it (*P* > 0.05).

Table 7 shows the simple and multiple linear regression model based on the bivariate analysis results for the overall HL score. After adjusting for the effect of other variables in the model, only the level of education and last time sought medical care variables still have a significant association with the overall HL score; despite that, the CI for the level of education includes 1, the adjusted *P* < 0.05, and adjusted R square is 0.065, which means that ~7% of the variation in the overall HL score is due to the model chosen.

**Table 7:** Simple and multiple linear regression analysis for the total HL score and variables which shows significant association in bivariate analysis among the adult general population in Qatar 2022.

**Explanatory variables**	**Un-Adjusted analysis**				**Adjusted analysis**			
	**B**	***P*** **-value**	**95.0% CI**		**B**	***P*** **-value**	**95.0% CI**	
			**Lower bound**	**Upper bound**			**Lower bound**	**Upper bound**
Nationality (Qatari)	0.786	**0.009** ^*^	**0.194**	**1.379**	0.321	0.669	−1.155	1.798
Age categories (≥ 61 years)	−0.211	0.146	−0.496	0.073	−0.057	0.800	−0.495	0.382
Income per QAR per Month (< 10,000 QAR)	0.077	0.431	0.114	0.268	0.107	0.411	−0.149	0.364
Education level (No formal education)	0.857	**< 0.001** ^*^	**0.603**	**1.112**	0.825	**< 0.001** ^*^	**0.427**	**1.223**
Spouse's education level (No formal education)	0.177	**0.046** ^*^	**0.003**	**0.351**	−0.159	0.330	−0.480	0.162
Father's educational level (No formal education)	0.293	**< 0.001** ^*^	**0.144**	**0.442**	0.087	0.578	−0.219	0.392
Mother's educational level (No formal education)	0.223	**0.003** ^*^	**0.076**	**0.370**	−0.186	0.235	−0.492	0.121
Migration status (non-migrants)	−0.824	**0.003**	**−1.177**	**−0.277**	−0.654	0.323	−1.952	0.644
Living with family (Yes)	−0.910	**0.008** ^*^	**−1.576**	**−0.240**	−0.936	0.051	−1.875	0.003
Self-rating of health status (I do not know)	0.178	0.258	−0.131	0.488	0.071	0.711	−0.303	0.444
Last time contacted medical care (within last year)	−0.274	**0.011** ^*^	**−0.484**	**−0.64**	−0.348	**0.019** ^*^	**−0.640**	**−0.057**

^*^P < 0.05.

## 4 Discussion

The main objective of this study is to find out the levels and prevalence of HL, and its competencies in Qatar as an important, under-studied intermediate modifiable factor to achieve better health outcomes. Moreover, the study addressed a hypothesized set of HL determinants as important means of HL interventions to minimize inequities in healthcare and support vulnerable groups. The validated AAHLS tool was used, but also, as recommended by the tool developers, the researcher tested for its internal consistency and Cronbach's alpha and yielded 0.51, indicating a poor internal consistency compared to the validation study. The variation in Cronbach's alpha can be justified by the variation in the sample size of the two studies, and the validation study sample size was 146, while our sample size valid for calculating Cronbach alpha was 766 (31). In addition, the researcher tested for construct validity, and it was good, as reflected by the positive correlation between the subscale scores and the total HL scale score with a *P* < 0.001, which was comparable to the original validation study (28).

### 4.1 Prevalence of HL

The results show that nearly half of the study population have an adequate HL level (41.5%), while approximately one-third (29.2%) have a low HL level and a high HL level (29.3%). Although the study is the first of its kind in Qatar, the results are comparable to the results of another study done among pregnant women in Qatar by Naja et al., which found that 54.6% of pregnant women have adequate HL levels (22). In comparison to other GCC countries, the study findings vary by almost 15% from the findings of the HL research done in Saudi Arabia by Al-Mubarak et al., which shows that 46% of the study respondents have low HL (10). It is also inconsistent with the second study from Saudi Arabia conducted by M. Abdel-Latif et al., which found that 57.4% of the Saudi study participants have inadequate HL (32), and a third study conducted in UAE by Nair et al. found that over 60% of the population surveyed possessed inadequate HL (33). Both the second and third studies reported almost double the prevalence of low HL the researcher found in Qatar. An important fact to consider that could justify our study results variation from other studies from the region is the limitation of the comparison when discussing HL as an evolving concept; due to the variation in the measurement tools used in different studies, most tools focus on functional HL (3).

Globally, a study covering the European Union member states conducted by Baccolini et al. showed that the pooled prevalence of low HL ranged from 27% to 48% depending on the literacy assessment method applied, which is not different from the study findings (4). In the United States, a systematic review of the prevalence of HL conducted by Paasche-Orlow et al. concluded that the weighted prevalence of low HL was 26%, which is also comparable to the study findings of 29.2% (5). In southeast Asia, a systematic review done by Rajah et al. found that the overall prevalence of low HL varied considerably, with a mean of 55.3%, which is 16% higher than the prevalence of low HL in this study (6). In contrast, the study findings contradict the findings of a study done in Turkey by Yigitalp et al., which shows a prevalence of 80.6% of low HL among their study population (8). Apart from variation in the tools used to measure HL, the inherent disparities in the social determinants of health among the different populations around the world could hinder fair comparison and, more importantly, variation in the healthcare systems in different contexts and timings.

Concerning the HL domains, the findings reveal that the higher prevalence of adequate HL is for communicative HL, followed by empowerment, critical HL, and functional HL, which are comparable to the findings of a similar study done in Bahrain using the same tool, whose findings is that the mean item scores were higher for empowerment, followed by communicative HL, suggesting confidence in these areas compared to critical and functional HL which had lower scores (11).

### 4.2 Determinants of HL

The study found an association between HL overall and subscale scores and the sociodemographic factors; Age, ethnicity, nationality, participant's level of Education, spouse, father and mother education level, income, migration status, and living with family were the variables with significant association. The older age group was associated with low overall HL, functional HL, and critical HL mean scores. These findings are comparable to the findings of other HL studies (5–7, 10, 11, 33). In contrast, despite what is proved by many other studies (7, 11, 16), gender does not prove to be associated with any of the HL domains in this study, which is the same finding as the United States HL studies (5). As addressed by the literature, the study proved that there is an association between HL scores and ethnicity/nationality (5, 12), Arab ethnicity, and Qatari as nationals are associated with low functional and communicative HL scores. Participant's low level of education is associated with low functional HL, critical HL, and overall HL scores, and these findings go with other HL evidence from the Eastern Mediterranean region (7), United States (5), and South East Asia (6). Despite that, the study does not show any association between the participant's occupation and HL scores. Unlike other HL review findings (12), it shows a consistent association between the low level of income < 10,000 QAR (< 2,740 USD) per month and low communication and overall HL scores, which is comparable to other HL research findings (6, 10, 12, 15). The language measured as the mother language does not prove to have an association with HL scores in this study. Unlike the finding of a review on HL from the West (14), our finding could be explained by the bilingual nature of Qatar's health system (Arabic and English). However, we cannot ignore our study limitation of excluding non-Arabic, non-English speakers.

Socially, marital status does not prove to have an association with HL scores, which contradicts similar studies from nearby countries (10, 11). In contrast, a father's higher education level is associated with high functional HL and overall HL scores, which is the same finding of another study from Palestine done on university students (16). In addition, a spouse's higher education level is associated with high functional HL scores and overall HL scores, similar to the findings of other studies from Iran (15). Unsurprisingly, the study found the same associations for mothers' education levels. As discussed in a qualitative study conducted in the UK, the HL could be distributed through families and social networks (13). The study findings of non-migrants having lower functional and total HL scores compared to migrants are also reflected by Qatari nationals having low functional and communicative HL compared to non-Qatari. Moreover, the condition of living with family or not proved to be associated with the overall HL score, as those who are not living within the family have a low overall HL score.

For health-relevant factors, those who sought medical care within the last week have lower critical HL scores compared to those who sought medical care earlier, and those who sought medical care before 1 year have a lower overall HL score compared to those who sought medical care more recently. These findings are inconsistent with each other and the findings on the same question from the Bahrain study (11), which can be explained by the higher possibility of recall bias in this question. In contrast, those who do not know that they have a chronic disease or do not have lower critical HL and overall HL compared to those who know are consistent with Bahrain study's findings for some of the critical HL questions (11).

### 4.3 Study strengths and limitations

Our study was the first in Qatar to measure the HL levels among the general adult population and address its possible determinants. The data collection through phone interviews conducted by a well-trained data collector and using real-time data entry ensured validity and consistency. The tool that was used, the All Aspects of HL Scale (AAHLS), is quite comprehensive compared to other HL measurement tools, which mainly focus on the functional HL (28). It is also favorable to be administered at the community level and well-established and used by nearby countries such as Bahrain and Iraq.

The main limitations of this study were that it was only on Arabic and English, and the exclusion of other languages, especially Urdu (The third common language in Qatar), could introduce a major selection bias. Favorably, the researcher managed to translate the tool into Urdu, but he could not manage to get the approval to use the tool before the end of the data collection due to time constraints; the tool in Urdu is available for future use by other researchers if needed. Furthermore, the tool showed a low internal consistency reflected by Cronbach alpha of 0.51, which needs to be further adapted and validated to be reliable. Moreover, the sample size of 770 could result in an underrepresentation of the population as the researchers could not collect more data on non-respondents to address the possibility of selection bias as they were either out of reach or refused to participate in the study by any means.

### 4.4 Conclusion

In conclusion, our study provides good insights into the prevalence and determinants of HL in the state of Qatar. Despite the challenges in comparison due to the variability in HL measurement tools and the inherent differences in the underlying contexts, we conclude that, in Qatar, there is a good prevalence of adequate HL level among the general adult population. We found that the higher adequate levels are for communicative and empowerment domains, followed by critical and functional HL.

Despite that, the main determinants of the overall HL levels, proved by multivariate analysis, are level of education and last time of contact with medical care; and age, ethnicity, nationality, spouse, father and mother education level, income, migration status, and living with family play a role in determining the overall HL and HL competency levels in the community.

### 4.5 Recommendations

Based on our findings and conclusion, we recommend several interventions at different levels:first, to further enhance the community level of education to university and postgraduate levels as an important determinant of HL level and consequently better health outcomes; second, given the importance of contact with medical care, to adopt a simpler approach to patient communication and information sharing in healthcare settings; and third, to consider the different possible determinants of HL while providing healthcare and build an HL-responsive health service to ensure health equity and better health outcomes.

### 4.6 Further research

Standardization of HL measurement tools is a requirement to ensure comparability between groups and, more importantly, over time for the same group. Further studies covering those who only speak other than Arabic and English languages need to be carried out to ensure inclusiveness in decision-making and equity in healthcare provision. An HL measurement tool with an established scoring mechanism is required to further ensure the validity of the results. Moreover, although the questionnaire has been validated for nearby countries such as Bahrain and Iraq, it should be validated for Qatar in future studies to ensure the results are reliable.

## Data availability statement

The raw data supporting the conclusions of this article will be made available by the authors, without undue reservation.

## Ethics statement

The studies involving humans were approved by Institutional Review Board (IRB) at Hamad Medical Corporation. The studies were conducted in accordance with the local legislation and institutional requirements. The Ethics Committee/Institutional Review Board waived the requirement of written informed consent for participation from the participants or the participants' legal guardians/next of kin because verbal informed consent was obtained from the participants and recorded as the data collection was via telephone interviews due to the COVID-19 pandemic.

## Author contributions

SA: Conceptualization, Data curation, Formal analysis, Funding acquisition, Investigation, Methodology, Project administration, Resources, Software, Validation, Visualization, Writing – original draft, Writing – review & editing. VK: Supervision, Writing – review & editing. MA: Data curation, Project administration, Writing – review & editing. IB: Supervision, Writing – review & editing.

## References

[1] Nutbeam D Lloyd JE. Understanding and responding to health literacy as a social determinant of health. Annu Rev Public Health. (2020) 42:159–73. 10.1146/annurev-publhealth-090419-102529 33035427

[2] Nutbeam D. Health literacy as a public health goal: a challenge for contemporary health education and communication strategies into the 21st century. Health Promot Int. (2000) 15:259–67. 10.1093/heapro/15.3.259

[3] Nutbeam D. Health literacy as a population strategy for health promotion. Jpn J Heal Educ Promot. (2017) 25:210–22. 10.1177/1757975918814436

[4] Baccolini V Rosso A Di Paolo C Isonne C Salerno C Migliara G. What is the prevalence of low health literacy in European Union Member States? A systematic review and meta-analysis. J Gen Intern Med. (2021) 36:753–61. 10.1007/s11606-020-06407-8 33403622 PMC7947142

[5] Paasche-Orlow MK Parker RM Gazmararian JA Nielsen-Bohlman LT Rudd RR. The prevalence of limited health literacy. J Gen Intern Med. (2005) 20:175. 10.1111/j.1525-1497.2005.40245.x 15836552 PMC1490053

[6] Rajah R Hassali MAA Murugiah MK. A systematic review of the prevalence of limited health literacy in Southeast Asian countries. Public Health. (2019) 167:8–15. 10.1016/j.puhe.2018.09.028 30544041

[7] Wikkeling-Scott LF Ajja RJY Rikard RV. Health literacy research in the Eastern Mediterranean Region: an integrative review. Int J Public Health. (2019) 64:523–33. 10.1007/s00038-018-01200-1 30815736

[8] Yigitalp G Bayram Deger V Çifçi S. Health literacy, health perception and related factors among different ethnic groups: a cross-sectional study in southeastern Turkey. BMC Public Health. (2021) 21:1. 10.1186/s12889-021-11119-7 34112137 PMC8194111

[9] Nair SC Satish KP Sreedharan J Muttappallymyalil J Ibrahim H. Letter to the editor: improving health literacy critical to optimize global telemedicine during COVID-19. Telemed J E Health. (2020) 26:1325. 10.1089/tmj.2020.0175 32463318

[10] Almubark R Basyouni M Alghanem A Althumairi N Alkhamis D Alharbi LS. Health literacy in Saudi Arabia: implications for public health and healthcare access. Pharmacol Res Perspect. (2019) 7:1–10. 10.1002/prp2.514 31397117 PMC6687660

[11] Asokan G V Yusuf MYA Kirubakaran R Albadwi AMM Alwardi AE Saad AE. Levels and determinants of health literacy in Bahrain's community context. Oman Med J. (2020) 35:1–25. 10.5001/omj.2020.88 33204521 PMC7642644

[12] Stormacq C Van Den Broucke S Wosinski J. Does health literacy mediate the relationship between socioeconomic status and health disparities? Integrative review. Health Promot Int. (2019) 34:E1–17. 10.1093/heapro/day062 30107564

[13] Edwards M Wood F Davies M Edwards A. “Distributed health literacy”: longitudinal qualitative analysis of the roles of health literacy mediators and social networks of people living with a long-term health condition. Health Expect. (2015) 18:1180–93. 10.1111/hex.12093 23773311 PMC5060848

[14] Ingleby D. Acquiring health literacy as a moral task. Int J Migr Heal Soc Care. (2012) 8:22–31. 10.1108/17479891211231383

[15] Ansari H Almasi Z Ansari-Moghaddam A Mohammadi M Peyvand M Hajmohammadi M. Health literacy in older adults and its related factors: a cross-sectional study in Southeast Iran. Heal Scope. (2016) 5:1. 10.17795/jhealthscope-37453

[16] Sarhan MBA Fujii Y Kiriya J Fujiya R Giacaman R Kitamura A. Exploring health literacy and its associated factors among Palestinian university students: a cross-sectional study. Health Promot Int. (2021) 36:854–65. 10.1093/heapro/daaa089 33141166 PMC8384377

[17] Berkman ND Sheridan SL Donahue KE Halpern DJ Crotty K. Low health literacy and health outcomes: an updated systematic review. Ann Intern Med. (2011) 155:97–107. 10.7326/0003-4819-155-2-201107190-00005 21768583

[18] Peterson PN Shetterly SM Clarke CL Bekelman DB Chan PS Allen LA. Health literacy and outcomes among patients with heart failure. JAMA. (2011) 305:1695–701. 10.1001/jama.2011.512 21521851 PMC4540335

[19] Ministry of Public Health Q. Ministry of Public Health - National Health Strategy 2018–2022. Available online at: https://www.moph.gov.qa/english/strategies/National-Health-Strategy-2018-2022/Pages/default.aspx (accessed May 12, 2022).

[20] Ministry of Public Health Q. Ministry of Public Health - Qatar Public Health Strategy 2022–2017. Available online at: https://www.moph.gov.qa/english/strategies/Supporting-Strategies-and-Frameworks/QatarPublicHealthStrategy/Pages/default.aspx (accessed May 12, 2022).

[21] Elyamani R Naja S Al-Dahshan A Hamoud H Bougmiza MI Alkubaisi N. Mental health literacy in Arab states of the Gulf Cooperation Council: a systematic review. PLoS ONE. (2021) 16:1. 10.1371/journal.pone.0245156 PMC7790272 33411793

[22] Naja S Elyamani R Al Ibrahim A Al Kubaisi N Itani R AbdulRouf P. ‘The newest vital sign among pregnant women attending women wellness and research Centre in Qatar: a cross-sectional study.' *BMC Pregnancy Childbirth*. (2021) 21:1. 10.1186/s12884-021-03542-w 33478419 PMC7819321

[23] Schoenbach K Khaled SM Wartella EA Saeed M. Health information and monitoring among Qatari adolescents 2017: an overview. In: Social Sciences, Arts and Humanities. (2019). Qatar: Northwestern University.

[24] Saleh OS Ahmed N. Usability of web tools and its impact on healthcare literacy in Qatar. J Qual Heal Care Econ. (2021) 4:1. 10.23880/jqhe-16000236

[25] Planning Statistics Authority Q. Topics Listing. Available online at: https://www.psa.gov.qa/en/statistics1/pages/topicslisting.aspx?parent=Population&child=Population (accessed May 12, 2022).

[26] OpenEpi. OpenEpi - Toolkit Shell for Developing New Applications. Available online at: https://www.openepi.com/SampleSize/SSPropor.htm (accessed May 12, 2022).

[27] ResearchGate. How to Adjust Sample Size for Non-Response in Cross Sectional Studies? | ResearchGate. Available online at: https://www.researchgate.net/post/How-to-adjust-sample-size-for-non-response-in-cross-sectional-studies (accessed April 18, 2023).

[28] Chinn D McCarthy C. All Aspects of Health Literacy Scale (AAHLS): Developing a tool to measure functional, communicative and critical health literacy in primary healthcare settings. Patient Educ Couns. (2013) 90:247–53. 10.1016/j.pec.2012.10.019 23206659

[29] Hatamleh J Huff M. Health literacy of Iraqi immigrant adults: pilot study. Int J Healthc. (2016) 2:121. 10.5430/ijh.v2n1p121

[30] Doane DP Seward LE. Measuring skewness: a forgotten statistic? J Stat Educ. (2011) 19:2. 10.1080/10691898.2011.11889611

[31] Bujang MA Omar ED Baharum NA. A review on sample size determination for cronbach's alpha test: a simple guide for researchers. Malays J Med Sci. (2018) 25:85–99. 10.21315/mjms2018.25.6.9 30914882 PMC6422571

[32] Abdel-Latif MMM Saad SY. Health literacy among Saudi population: a cross-sectional study. Health Promot Int. (2019) 34:60–70. 10.1093/heapro/dax043 28973389

[33] Nair SC Sreedharan J Satish KP Ibrahim H. Health literacy in a high income Arab country: a nation-wide cross-sectional survey study. PLoS ONE. (2022) 17:1–11. 10.1371/journal.pone.0275579 36197929 PMC9534436

