# Corrigendum: Prevalence and determinants of health literacy among the adult population of Qatar

**DOI:** 10.3389/fpubh.2024.1472003

**Published:** 2024-10-04

**Authors:** Salma Ahmed, Vahe Kehyayan, Mariam Abdou, Iheb Bougmiza

**Affiliations:** ^1^Department of Community Medicine, Hamad Medical Corporation, Doha, Qatar; ^2^College of Business Management, University of Doha for Science and Technology, Doha, Qatar; ^3^Department of Community Medicine, Primary Healthcare Corporation, Doha, Qatar

**Keywords:** health literacy, health knowledge, prevalence, determinants, Middle East, public health, questionnaire

In the published article, there was an error in the Funding statement. It originally stated “The author(s) declare financial support was received for the research, authorship, and/or publication of this article. This study was funded by the Hamad Medical corporation. Open Access funding is provided by the Hamad Medical corporation.”

The correct Funding statement appears below:

